# QUALITY OF LIFE OF OLDER INFORMAL CAREGIVERS IN SWEDEN: CAREGIVER PROFILES AND RELATED FACTORS

**DOI:** 10.1093/geroni/igae098.1762

**Published:** 2024-12-31

**Authors:** Mariam Kirvalidze, Maider Mateo-Abad, Alba Ayala, Amaya Bernal-Alonso, Maria João Forjaz, Carmen Rodríguez-Blázquez, Amaia Calderón-Larrañaga

**Affiliations:** Karolinska Institutet, Stockholm, Stockholms Lan, Sweden; Biogipuzkoa Health Research Institute, San Sebastian, Pais Vasco, Spain; Instituto de Salud Carlos III, Madrid, Madrid, Spain; Universidad Nacional Educación a Distancia, Madrid, Madrid, Spain; National Centre of Epidemiology, Carlos III Institute of Health, Madrid, Madrid, Spain; National Centre of Epidemiology, Carlos III Institute of Health, Madrid, Madrid, Spain; Karolinska Institutet, Stockholm, Stockholms Lan, Sweden

## Abstract

Older adults are increasingly stepping into informal caregiving roles, primarily due to the inadequacy of formal care services to meet their growing demands. We aimed at identifying profiles of older caregivers according to their quality of life (QoL), and describing the factors characterizing them. A cross-sectional analysis of a cohort study was conducted, including informal caregivers from the Swedish National Study on Aging and Care in Kungsholmen. The study included a total of 1382 observations, corresponding to 994 caregivers aged 60 and above, from waves 1 (2001) to 5 (2013). Multiple Correspondence Analysis combined with cluster analysis were used to obtain the caregiver profiles according to the items of SF-12 QoL instrument. Multinomial regression was performed to determine which demographic, clinical and psychosocial factors were related to belonging to each profile. Three caregiver profiles were identified according to the physical and mental health domains of their QoL. People with good QoL (58%) had better mental and physical health status, and reported a lower caregiver burden. People with intermediate QoL (35%) were older than those in the other profiles and, along with the group with poor QoL (7%), had worse health status and often received care themselves. The poor QoL group was also characterized by higher prevalence of depression and perception of burden, and lower levels of social support. Different modifiable factors are associated with the QoL of older informal caregivers, especially in the mental health domain, and could thus be the target of future preventive interventions.

